# Tribological Characterization of Micron-/Nano-Sized WC-9%Co Cemented Carbides Prepared by Spark Plasma Sintering at Elevated Temperatures

**DOI:** 10.3390/ma12060920

**Published:** 2019-03-20

**Authors:** Saleh Al Wohaibi, Abdul Samad Mohammed, Tahar Laoui, Abbas Saeed Hakeem, Akeem Yusuf Adesina, Faheemuddin Patel

**Affiliations:** 1 Department of Mechanical Engineering, King Fahd University of Petroleum and Minerals, Dhahran 31261, Saudi Arabia; g200641640@kfupm.edu.sa (S.A.W.); samad@kfupm.edu.sa (A.S.M.); faheemmp@kfupm.edu.sa (F.P.); 2 Department of Mechanical and Nuclear Engineering, University of Sharjah, Sharjah 27272, UAE; 3 Center of Excellence in Nanotechnology, King Fahd University of Petroleum and Minerals, Dhahran 31261, Saudi Arabia; ashakeem@kfupm.edu.sa; 4 Center of Research Excellence in Corrosion, Research Institute, King Fahd University of Petroleum and Minerals, Dhahran 31261, Saudi Arabia; g201101750@kfupm.edu.sa

**Keywords:** tungsten carbide-cobalt, cemented carbide, powder processing, spark plasma sintering, nanomaterials, wear, elevated temperature

## Abstract

The present study investigates the high temperature tribological performance of spark plasma sintered, nano- and micron-sized tungsten carbide (WC) bonded by 9 wt.% cobalt (Co). The composites were fabricated using a two-step procedure of mixing followed by spark plasma sintering (SPS). Ball-on-disc wear tests were conducted at a normal load of 30 N, linear speed of 0.1 m/s under dry conditions and at three different temperatures (room temperature, 300 °C and 600 °C). Field emission scanning electron microscopy (FESEM), optical profilometry and energy dispersive X-ray (EDS) spectroscopy were used to analyze the surface morphology and the wear track area. At room temperature, it was observed that the nano-sized WC composites exhibited better wear resistance than the micron-sized WC composites. The wear resistance of the nano-sized samples declined significantly relative to that of the micron-sized samples with an increase in temperature. This decline in performance was attributed to the higher surface area of nano-sized WC particles, which underwent rapid oxidation at elevated temperatures, resulting in poor wear resistance. The wear rate observed at 600 °C for the micron-sized WC composites was 75% lower than that of the nano-sized cemented carbide. Oxidative wear was observed to be the predominant wear mechanism for both cemented carbide samples at elevated temperatures.

## 1. Introduction

Tungsten carbide-cobalt (WC-Co) cemented carbides are considered the best materials used in the hard metal industry. They have the following remarkable mechanical properties: High hardness, excellent high temperature strength, a high elastic modulus, good wear resistance, corrosion resistance and excellent chemical stability at high temperatures. Cemented carbides have been extensively used for cutting tool tip applications, which require high levels of wear resistance at elevated temperatures, as the tool-tip is subjected to high temperatures during machining processes. Therefore, it is crucial to develop an understanding of the wear behavior of these carbides at room and elevated temperatures.

As noted above, cemented carbides are generally appropriate materials to use for high speed machining and dry cutting due to their ability to retain their hardness even at elevated temperatures. Temperatures of roughly 600 °C are usually encountered by tools during high speed machining or dry cutting, and in turn, properties such as wear and friction at the tool-chip interface become significant and can affect the overall tool life [1]. Various studies have been conducted to evaluate the tribological behavior of WC-Co carbides at room temperature, and a few have been conducted at elevated temperatures, as summarized below.

B. Yaman and H. Mandal [2] studied the tribological behavior of cemented carbides prepared by spark plasma sintering (SPS) using ball-on-disc wear tests under dry conditions and at room temperature, revealing the outstanding features of SPS sintered WC-Co composites compared to conventional sintered ones (typically hot pressed) in terms of better wear properties for tribological applications. They found a 45% improvement in wear rate of SPSed WC-Co (3.37 × 10^−9^ mm^3^/N-m) compared to the conventional one (6.08 × 10^−9^ mm^3^/N-m). They observed dominant wear mechanisms to be microcracking, grain pull out and formation and spalling of tribochemical layers. Espinosa et al. [3] carried out friction and wear tests using a ball-on-disc test with high loads (40 N to 60 N) on WC-12 wt.% Co composites obtained from nanocrystalline mixtures at room temperature. They studied the effect of grain growth inhibitors such as chromium carbide (Cr_3_C_2_) and vanadium carbide (VC) on wear properties. They observed significant enhancement of wear resistance in the order of 90% for VC with a wear rate of 18.9 × 10^−7^ mm^3^/N-m in the conventional material as compared to of 2.2 × 10^−7^ mm^3^/N-m with 1% VC. J. Perso et al. [4] investigated the tribological properties of WC-Co using block-on-ring configurations with different loads, speeds and cobalt contents at room temperature. They found that the wear mechanisms were predominantly dependent on the applied load and material composition. Predominant mechanisms included the removal of the cobalt binder, fracture of intergranular boundaries and fragmentation of tungsten carbide particles. Deng Jianxin et al. [1] studied the wear and friction behaviors of cutting tool materials made of cemented carbide with varied WC grain sizes (0.6 to 2.2 μm) under room to 600 °C test temperature. They reported that the friction coefficient decreased whereas the wear rate increased with increasing test temperature and sliding speeds. Y. Pérez Delgado et al. [5] studied the wear and friction behavior of WC-10%Co(Cr/V) alloys with different surface finish produced by grinding and wire-EDM process at 25 °C and 400 °C test temperatures. They observed that wire-EDM processed samples had a 3.1 times higher wear rate compared to ground samples and the friction coefficient was also observed to be higher at 0.75 ± 0.1 for wire EDM samples as compared to 0.71 ± 0.8 for ground samples, however, the temperature of the test conducted was observed to have no important effect on the wear rate. E. Marui et al. [6] investigated the high temperature (400 °C) wear characteristics of WC-Co cemented carbides synthesized with a small amount (less than 1%) of Ti and Ta. They reported that the test temperature of the contacting surface greatly affected the wear. They observed that during earlier stages of wear, Co and Ti, melted by friction heating, and transferred to the disc specimen surface because of their low melting point thereby weakening the binding force between WC and TaC. This led to the transferring of these elements to the disc specimen surface, causing further wear of the WC-Co alloy. Hui Zhang et al. [7] observed the wear behavior of cemented carbides at elevated temperatures and found that the wear rate increased with increasing operating temperature but speed did not have a strong influence on the wear rate like the operating temperature did. T. Kagnaya et al. [8] performed measurements of wear parameters at high sliding speeds to mimic high speed cutting tool conditions and better understand the wear mechanisms of WC-Co pins. They reported that WC-Co materials exhibited a variety of wear mechanisms including abrasion, adhesion, transgranular WC micro-cracking and WC/WC debonding. The friction coefficient was observed to decrease from 0.63 to 0.4 with an increase in sliding speeds from 60 m/min to 600 m/min, whereas the wear rate increased with higher sliding speeds with a linear relationship between dissipated energy and wear loss. At higher sliding speeds additional wear mechanism of material transfer from the disk to pin was observed. Wenbin Ji et al. [9] studied the effect of sliding speed and load on wear behavior of cemented carbide disk sliding against Al_2_O_3_ and Si_3_N_4_ balls. They observed that the friction coefficient was not affected much by the sliding speeds for both Al_2_O_3_ and Si_3_N_4_. The wear rate was found to be highly dependent on sliding speeds and loads during sliding against Al_2_O_3_ ceramics, however, a higher friction coefficient but a lower and constant wear rate was observed for Si_3_N_4_ ceramics. Recently, Yulin Liu et al. [10] studied the tribological behavior of pure tungsten carbide in a temperature range from 25 to 800 °C under vacuum and air. They observed that the tribological properties were significantly affected by the atmosphere. Pure tungsten carbide could maintain good phase stability in the selected range of temperatures, however, it was predominantly affected by oxidation under air and highest oxidation took place in a temperature range of 500 °C to 550 °C. Verma et al. [11] studied the effect of the addition of TaC and Ni-Co on tribological properties of TiCN-based cermets sliding against cemented carbide hard counterbody. They reported that the size and contiguity of the ceramic phase, mean free path of binder phase, contact temperature and the ionic potential difference between oxide debris significantly affected the wear and frictional characteristics of TiCN-WC Ni/Co cermets. Liu et al. [12] compared the tribological behavior of spark plasma sintered WC-10 wt% Co and WC-10 wt% Fe_3_Al hard metals. They observed that the friction coefficient of both WC-10 wt% Co and WC-10 wt% Fe_3_Al decreased with increasing sliding velocity under different applied loads. However, the hardness and wear resistance of WC-Fe_3_Al was found to be much higher than that of WC-10 wt% Co. This improvement was attributed to the formation of Al_2_O_3_. Ramirez et al. [13] studied the diffusion wear behavior of WC-10 wt% Co tools during dry machining of commercial titanium alloys. A diffusion couple between the tool and the machined alloy revealed an early formation of the titanium carbide layer at the interface with more concentration at the titanium alloy. A decreasing WC gradient was observed on the tool side and diffusion of Co, C and W was observed on the alloy side, with W diffusing to a lower depth compared to C and Co. Mao et al. [14] studied the tribological behavior of cBN-WC-10 wt% Co composites subjected to the dry reciprocating sliding wear against SiC ceramic ball at an ambient temperature. They reported that the addition of cBN particles improved the wear resistance of cBN-WC-10Co composites, which was attributed to cBN particles in the composites acting as micro cutting edges. Presence of both abrasive wear and oxidation wear was observed with abrasive wear being the dominant mechanism. The particle size of cBN was found to affect the specific wear rate with small particle sizes in cBN-WC-10Co composites leading to a reduced specific wear rate at normal loads below 50 N whereas large particle sizes showed better wear resistance when the normal load was continuously increased. Muthuraja et al. [15] studied the wear behavior of a tungsten carbide cutting tool fabricated with and without solid lubricant (WC-10Co-5CaF2 and WC-10Co) during machining of AISI 1020 steel material under a dry condition. Tungsten carbide with solid lubricant generated 20–40% less cutting force and showed 15–18% reduction in flank wear when compared to the cutting tool without solid lubricant.

From the literature review above, very few studies have addressed the effect of particle size and test temperature environment. Thus, the focus of the present study was to evaluate the wear rate of WC-9%Co cemented carbide possessing two WC particle sizes (micron and nano size), compare their performance at different temperatures, room temperature (RT); 300 °C and 600 °C, under dry conditions, and underpin the underlying wear mechanisms.

## 2. Experimental Procedures

### 2.1. Raw Materials and Sample Fabrication

The starting powder materials used in this study were tungsten carbide (WC) with an average particle size of 3.5 µm as a hard base material and cobalt (Co) with an average particle size of 1.5 µm as a soft binding material. Both materials were procured from William Rowland Limited, UK.

Samples of cylindrical geometry (20 mm in diameter by 6 mm in height) were fabricated using a two-step process of high energy ball milling for the uniform mixing of powders followed by spark plasma sintering (SPS) for consolidation.

### 2.2. Ball Milling Procedure

All milling and mixing operations were carried out using a high-energy ball mill (Model HD-01/HDDM-01 Lab Attritor, UNION PROCESS, Akron, OH, USA). The as-received WC powder of 3.5 µm was milled to create the required nano-sized WC powder after 5 h at 2000 rpm using zirconia balls of 0.625 mm in diameter. A powder-to-ball ratio of 1:20 was used in a medium of ethanol. During milling, powder samples were taken from a vial at different intervals for analysis to determine the size through field emission scanning electron microscopy (FESEM, Lyra 3, Tescan, Czech Republic) until an average particle size of 100 nm was obtained. An analysis was carried out to determine changes in the morphology of the powders and microstructure as an effect of the milling time using a secondary electron mode at an accelerating voltage of 30 kV. Energy dispersive X-ray spectroscopy (EDS, Oxford Inc., UK) was used to confirm the compositions and purity of the samples.

To obtain a homogeneous mixture of WC-9% Co for both micron- and nano-sized mixtures, WC balls of 6 mm in size subjected to a rotational speed of 200 rpm were used in the above described ball mill. The ball-to-powder ratio and the time were set to 1:5 and one hour for the micron-sized mixture and to 1:10 and three hours for the nano-sized mixture, respectively. In a previous study, it was observed that the addition of 9 wt.% cobalt to cemented carbide produced hard and dense samples for both sizes of WC prepared by spark plasma sintering (SPS) [16]. After mixing, the powders were dried at room temperature for 24 h to ensure the complete evaporation of ethanol, and they were successively used to prepare dense samples.

### 2.3. Spark Plasma Sintering (SPS) Procedure

Spark plasma sintering (SPS) (HP D-50 type, FCT System, Rauenstein, Germany) was carried out using graphite dies in a vacuum at a 100 °C/min heating rate with 10 min of holding time. The sintering parameters were optimized by preparing numerous samples and then analyzing them in terms of densification, porosity and microstructure features. The optimized sintering pressure and temperature for the micron-sized mixture of WC-9%Co was 45 MPa and 1200 °C. For the nano-sized mixture of WC-9% Co, the optimized sintering pressure and temperature was 50 MPa and 1250 °C, respectively.

### 2.4. Surface Preparation and Sample Morphology

All of the sintered samples were mounted using Buehler transoptic powder with a polymer pressed in a hot press (Evolution, IPA 40 Remet, Bologna, Italy) at 200 °C for 30 min. However, we must note that the polymer was removed when conducting wear tests at elevated temperatures. The samples were ground and polished by discs (Buehler Magno, Lake Bluff, IL, USA) using 125, 74, 40, 20 and 10 µm diamond grinding sheets successively while using water as a medium. This was followed by polishing using a Buehler diamond suspension of 3 µm particle size. Optical profilometer (ContourGT-K, Bruker, Billerica, MA, USA) based on light superposition according to interferometric principle was used to measure the surface roughness. The surface roughness was quantified using the amplitude parameter arithmetic mean (Ra), which defines the average deviation of all peaks and valleys of the profile from the mean line. Thus, the Ra value of micron-sized and nano-sized WC-9% Co samples was 0.11 ± 0.01 µm and 0.17 ± 0.02 µm, respectively.

Samples were coated with a thin layer of gold using a sputter coater (Model Q150T, Quorum Technologies, UK) for SEM imaging. A field emission scanning electron microscope (FESEM) was used to characterize the samples. The electron gun voltage was varied from 20–30 kV to obtain the best possible contrast. Both secondary and backscattered imaging modes were utilized in this study. An energy dispersive spectrometer accessory was of great help in linking the EDS phases with their corresponding morphologies.

### 2.5. Density and Hardness Measurements

The sintered samples were characterized to measure their density and hardness before wear testing. The density of the samples was measured using an MD-300S Alfa Mirage SG, resolution of 0.001 g/cm^3^ and capacity of 300 g, densimeter based on the Archimedes principle, and the Vickers hardness (HV30) was measured with a universal hardness testing machine (Zwick-Roell, ZHU250, Ulm, Germany) using a load of 30 kg. The optimized sintering parameters used in this study for spark plasma sintering (SPS) of both micron- and nano-sized WC-9% Co samples created dense and hard bulk samples. The observed density and hardness of the 3.5 µm WC-9% Co samples were measured as 13.77 (g/cm^3^) with 93.9% densification and 1411 (HV30), while the observed density and hardness of the 100 nm WC-9% Co samples were measured as 13.62 (g/cm^3^) with 92.8% densification and 1495 (HV30).

### 2.6. Wear Tests

Wear testing of samples was conducted using a tribometer (UMT-3, BRUKER, USA) with a ball-on-disc configuration at room and elevated temperature(s) using an alumina ball (6.3 mm diameter, HV = 1707) as the counterface. Wear tests were conducted at a normal load of 30 N, at a linear speed of 0.1 m/s under dry conditions and at three different temperatures (RT, 300 °C and 600 °C). A high temperature (up to 1000 °C) rotary chamber module of the UMT-3 tribometer was used for the high temperature test. The temperatures were set and allow to stabilize for about 10–15 min prior to commencing the test. The high temperature rotary chamber was insulated with refractory material to ensure that the test temperature was kept constant during testing. For the wear tests conducted at 600 °C, a sample fixture made of H-13 tool steel was fabricated to hold the samples. The high temperature module of a UMT-3 tribometer with an insulated chamber was used to ensure that the test temperature was kept constant during testing. The samples were cleaned with acetone and were properly dried before each wear test. Wear tests were carried out for a sliding distance of 500 m. After the wear tests, the wear track was examined under an optical profilometer (Contour GT-K Automated, BRUKER, USA) to measure the wear track depth and volume to calculate the specific wear rate. A minimum of three runs of wear tests for each condition was performed, and average values are reported. After every run, optical images of the counterface ball were taken to analyze the wear on the counterface ball during the test. Thermogravimetric analysis (TGA) of the samples was conducted at 600 °C in air using a TGA—STA 449F3—Jupiter (Netzsch, Germany), and then, a qualitative/quantitative X-ray diffraction (XRD) analysis was carried out using a MiniFlex II (Rigaku, Japan) to understand the wear mechanisms.

## 3. Results and Discussion

### 3.1. Surface Morphology Characterization and Mechanical Properties

**Figure 1:**
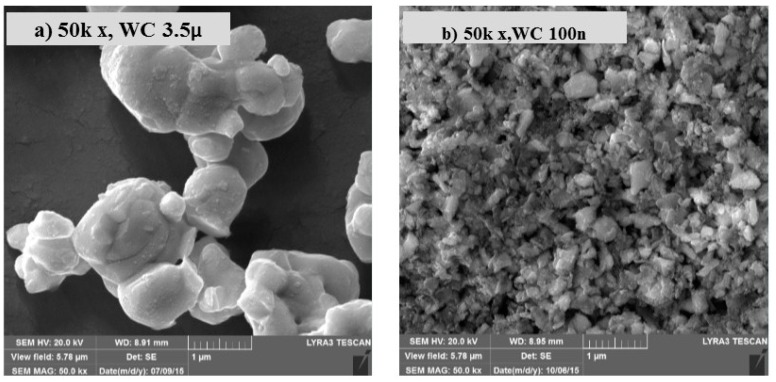
(**a**) FESEM image of the starting powder of WC, (**b**) SEM image of milled WC reaching an average size of 100 nm.

Figure 1a shows an FESEM image of the as-received tungsten carbide with an average particle size of 3.5 µm. Figure 1b shows the milled tungsten carbide, whereby a significant reduction in the particle size from 3.5 µm to approximately 100 nm can be observed.

**Figure 2:**
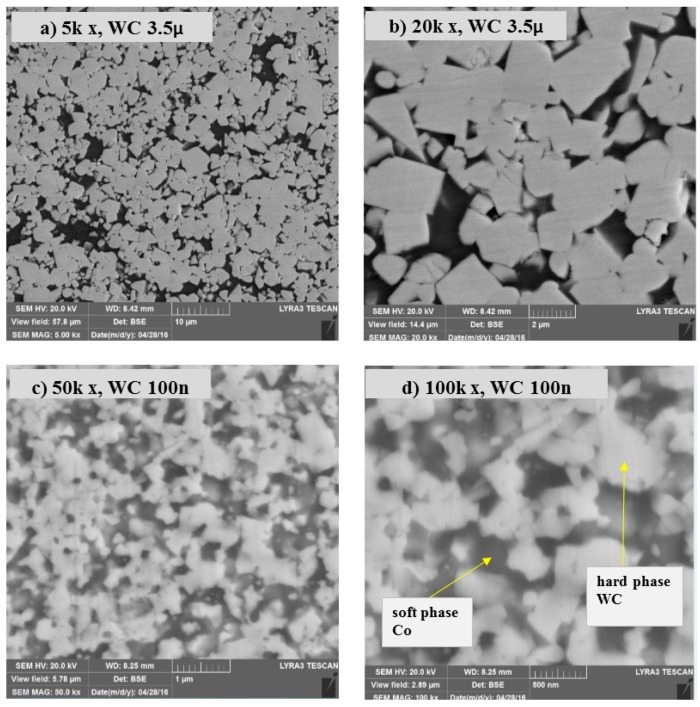
BSE images of samples sintered by SPS (**a**,**b**) 3.5 µm WC-9% CO at 45 MPa and 1200 °C and (**c**,**d**) 100 nm WC-9% Co at 50 MPa and 1250 °C.

From the backscattered emission (BSE) FESEM images collected after sintering, shown in Figure 2a,b at different magnifications (kX5, kX20) show that the cobalt was uniformly distributed within the WC in the micron-sized samples. Figure 2c,d shows the sintered nano-sized samples at different magnifications (kX50, kX100), in which cobalt was uniformly distributed with no agglomeration, showing that the mixing and sintering parameters of the samples used in this study produced a homogeneous mixture and dispersed cobalt. The uniform distribution of the soft phase (cobalt) within the hard phase (WC) was very significant, as the mechanical properties are known to be anisotropic in WC-Co due to the crystallographic structure of WC hard metals [17].

### 3.2. Effect of Temperature on the Tribological Properties of the Micron- and Nano-Sized WC Samples

**Figure 3:**
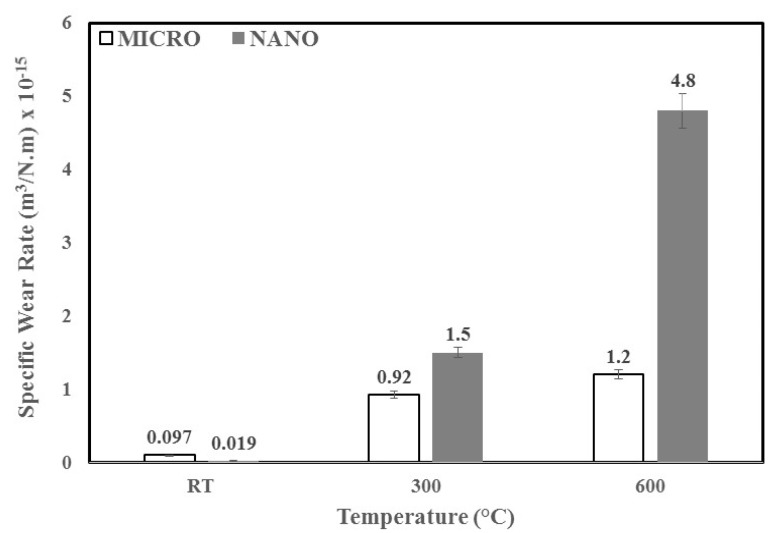
Variation in the specific wear rate with WC particle size and test temperature at a constant sliding velocity of 0.1 m/s and a normal load of 30 N under dry conditions.

The average wear rates observed at different temperatures (RT, 300 °C, 600 °C) for both the micron- and nano-sized WC samples are shown in Figure 3 for a normal load of 30 N and at a linear speed of 0.1 m/s. As can be observed from the figure, the specific wear rate shows an increasing trend for both WC samples with an increase in temperature. However, it was interesting to note that at higher temperatures (300 °C and 600 °C), the nano-sized WC samples showed higher wear rates than the micron-sized WC samples. At a temperature of 600 °C, the specific wear rate of the nano-sized WC samples increased by 99.6% relative to that observed at room temperature. This deterioration in the wear resistance of the nano-sized WC samples observed at elevated temperatures is attributed to the higher oxidation of the nano-sized WC particles resulting from the increase in surface area, which is also confirmed by the TGA and XRD characterization presented below.

**Figure 4:**
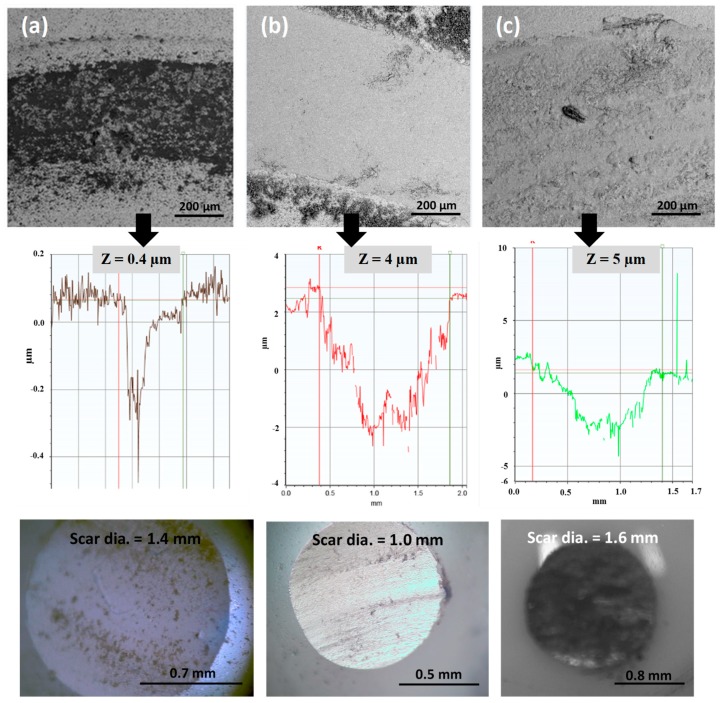
FESEM images, 2D profile plot showing the depth (Z) of the wear tracks and corresponding optical images of the counterface ball revealing the scar diameter of wear test conducted at (**a**) RT, (**b**) 300 °C and (**c**) 600 °C, for the micron-sized WC samples.

**Figure 5:**
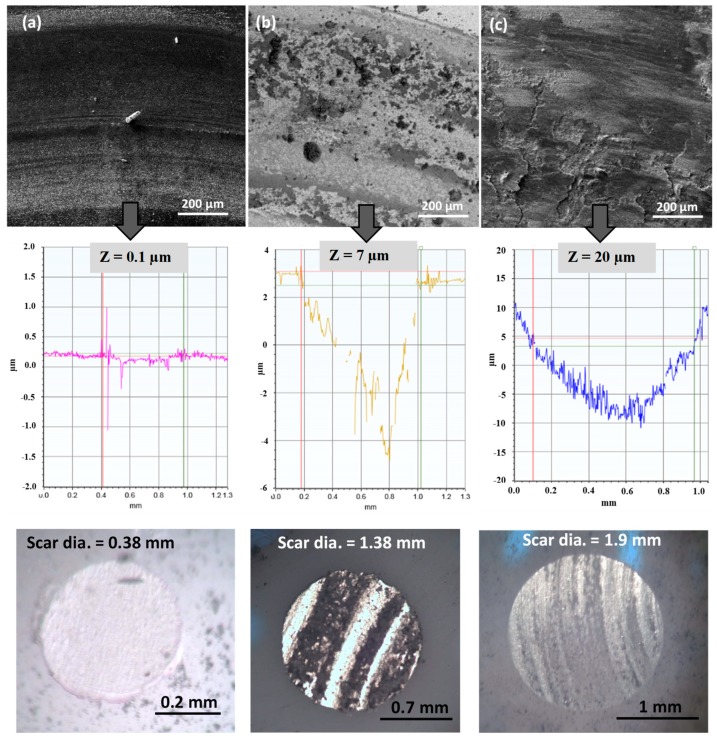
FESEM images, 2D profile plot showing the depth (Z) of the wear tracks and corresponding optical images of the counterface ball revealing the scar diameter of wear test conducted at (**a**) RT, (**b**) 300 °C and (**c**) 600 °C, for the nano-sized WC samples.

Figure 4 and Figure 5 show the wear track characteristics for micron- and nano-sized WC samples, respectively, at different temperatures of RT, 300 °C and 600 °C. Wear tracks were analyzed via FESEM imaging at X300 magnification followed by 2-D profiling using an optical profilometer and scar mark recording along the counterface ball using an optical microscope. In Figure 4 and Figure 5, both FESEM and profile images confirm that as the temperature increased, the wear rate showed an increasing trend at a linear speed of 0.1 m/s and a normal load of 30 N for both the micron- and nano-sized WC samples, as shown by the deeper wear tracks at 300 °C and 600 °C. The increased wear rates observed for both samples were attributed to the enhanced oxidation of WC particles at elevated temperatures, leading to a reduction in material wear resistance. However, the nano-sized WC samples showed higher wear rates than the micron-sized WC samples at elevated temperatures of 300 °C and 600 °C. This was mainly attributed to the higher oxidation rate of the nano-sized WC samples due to their larger surface area compared to that of the micron-sized WC samples, thus confirming that oxidative wear constitutes the main wear mechanism at higher temperatures. The formation of oxides at higher temperatures has a detrimental effect on the mechanical properties of WC-9% Co, leading to poor wear performance [18].

Figure 4 and Figure 5 also show optical images of the alumina counterface ball after wear tests conducted at different temperatures for the micron- and nano-sized WC samples. Cemented carbide debris was found to be transferred onto the alumina counterface, as observed from the optical images. As seen from the scar diameters on images of the counterface ball, the size of the scars increased with temperature when slid against both the micron- and nano-sized WC samples, signifying an increase in the wear rate with temperature in both cases. However, it must be noted that the scar mark diameter of the balls slid against the nano-sized WC samples was larger than that of the balls slid against the micron-sized WC samples at higher temperatures of 300 °C and 600 °C, signifying an increase in wear for the nano-sized WC samples at elevated temperatures.

However, at room temperature, the nano-sized WC samples performed better than the micron-sized WC samples in terms of both wear track depth and scar mark diameter. This better performance is attributed to the larger surface area of the nano-sized WC samples, leading to an increase in the effective area available for efficient bonding between the hard WC particles and the soft Co binder, hence rendering material pull-out difficult and leading to higher levels of wear resistance.

### 3.3. Wear Track Analysis and Characterization

**Figure 6:**
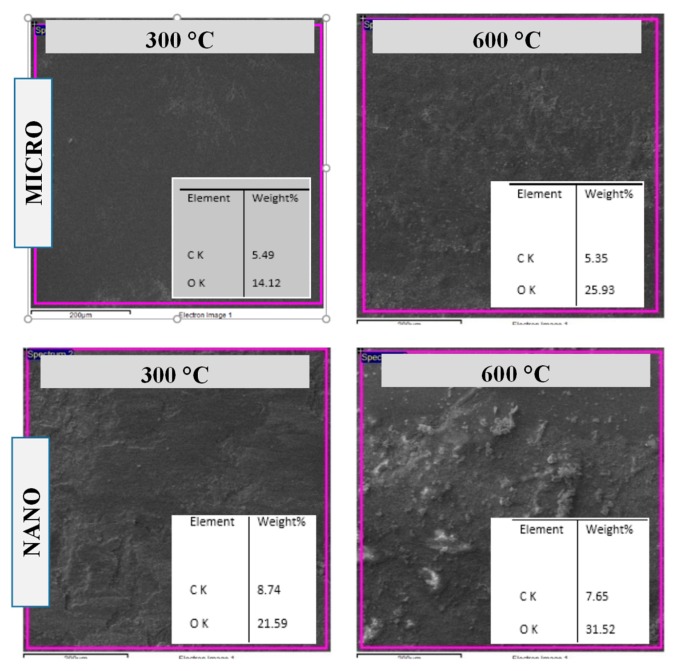
EDS analysis conducted on the wear tracks of the micron-and nano-sized WC samples after the wear test conducted at a load of 30 N, a speed of 0.1 m/s and at temperatures of 300 °C and 600 °C.

To further understand the underlying wear mechanisms and to evaluate why higher wear rates were found for the nano-sized WC samples compared to the micron-sized WC samples, an EDS analysis was conducted on the wear tracks of both sets of samples. Figure 6 shows FESEM/EDS spectra obtained for the wear tracks formed on both the micron- and nano-sized WC samples after the wear tests at temperatures of 300 °C and 600 °C. Interestingly, the formation of oxides on the surface can be clearly seen on the wear tracks of the samples after the wear tests conducted at elevated temperatures, as indicated by an increase in the intensity of the oxygen peak with increasing temperature. It was noted from the EDS analysis that the oxygen content in the wear track of the micron-sized WC samples increased from about 14.12 wt.% at 300 °C to about 25.93 wt.% at 600 °C. Similarly, for nano-sized WC samples the oxygen content in the wear track increased from about 21.59 wt.% at 300 °C to about 31.52 wt.% at 600 °C. This substantiates the observed raise in the oxidation rate with temperature. Moreover, we note that the oxygen contents observed at 300 °C and 600 °C are higher for the nano-sized WC samples (21.59 wt.% and 31.52 wt.%) than for the micron-sized WC samples (14.12 wt.% and 25.93 wt.%), suggesting an increase in the oxidation rate of the nano-sized WC samples at higher temperatures.

**Figure 7:**
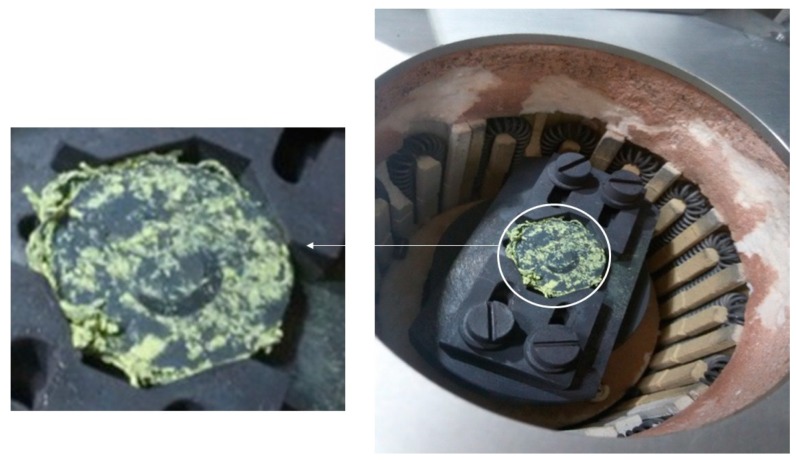
Digital image of the nano-sized WC-9% Co sample inside the heating chamber after the wear test was conducted at a normal load of 30 N, a speed of 0.1 m/s and temperature of 600 °C.

**Figure 8:**
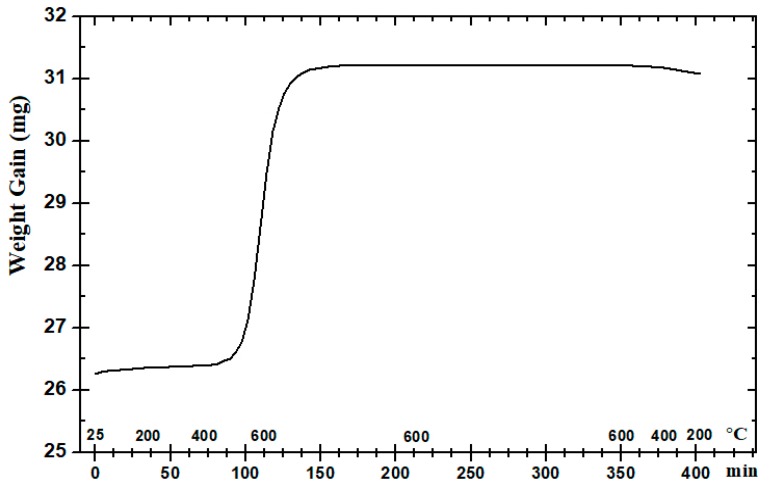
TGA results showing WC weight gain versus time up to a temperature of 600 °C.

**Figure 9:**
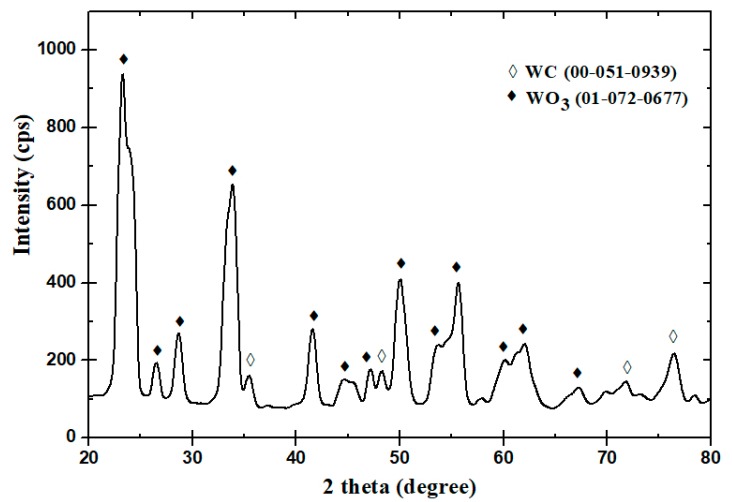
XRD spectrum of the WC sample, showing the intensity measured for the resulting compound after the TGA test in comparison to the database spectrum of tungsten oxide (WO_3_).

Figure 7 shows a nano-sized WC sample within the heating chamber of the UMT-3 tribometer immediately after the wear test conducted at 600 °C. Oxide layers have clearly formed on the surface of the sample. It has been observed in earlier studies that at higher temperatures, WC has a tendency to form a metal-oxide compound of tungsten oxide (WO_3_) [11]. Moreover, the oxide layer thickness has been found to increase considerably with increasing test temperature and in the presence of ultra-fine cemented carbides [19]. WO_3_ has also been found to be the dominant kind of oxide formed by WC hard metals at elevated temperatures with Co serving as the matrix with other inhibitors such as Ni and Cr [20]. Thus, to further confirm the formation of WO_3_, TGA and XRD analyses were conducted. Figure 8 shows the TGA results found for WC heated to 600 °C in air and clearly shows an increase in weight with increasing the temperature to 600 °C, suggesting oxidation and the formation of metal-oxide compounds. After the TGA analysis, the WC sample was subjected to XRD characterization to measure the formation of tungsten oxide (WO_3_). Figure 9 shows the XRD spectrum for the WC sample immediately after the TGA analysis. It confirms the formation of tungsten oxide (WO_3_) after matching with the database spectrum for WC (ICDD 00-051-0939) and tungsten oxide (ICDD 01-072-0677). The spectrum showed that most of the WC (~10%) was transferred into WO_3_ (~90%).

As can be seen from the above results regarding the different characterization techniques (EDS, TGA and XRD), the oxidation of WC leading to the formation of WO_3_ confirmed for both the micron-and nano-sized WC samples at the elevated temperatures of 300 °C and 600 °C, this indicated that the oxidative wear was the predominant wear mechanism at high temperatures [21,22,23]. The possible chemical reaction that would generate WO_3_ product can be given by Gibbs free energy equation [23]: WC + 9Co + 7O_2_ = WO_3_ + 9CoO + CO_2_. Moreover, the amount of oxides present was found to increase with an increase in temperature for both samples, with greater amounts of oxides found for the nano-sized samples than for the micron-sized samples. This was mainly attributed to the larger surface area of the nano-sized WC samples available for oxidation. Hence, in view of the above characterization results coupled with our examination of FESEM images of wear tracks after tests, it can be concluded that the wear mechanisms of both the micron- and nano-sized samples at elevated temperatures involve a combination of abrasive and oxidative wear, with oxidative wear playing a significant role, resulting in greater material loss.

## 4. Conclusions

- The sintering parameters used for spark plasma sintering (SPS) of micron- and nano-sized particles allowed for the formation of dense and hard bulk samples of WC-9% Co.
- At room temperature, it was observed that nano-sized WC exhibited better wear resistance than micron-sized WC, with a specific wear rate of 0.019 × 10^−19^ m^3^/N.m for nano and 0.097 × 10^−19^ m^3^/N.m for micron. This was attributed to the larger surface area of nano-sized WC particles, which facilitated binding between the hard phase (WC) and the soft binder (Co), leading to efficient load distribution.
- However, with an increase in temperature, the performance in terms of the wear resistance of the nano-sized samples declined significantly relative to that of the micron-sized samples exhibiting a specific wear rate of 1.5 × 10^−15^ m^3^/N.m for nano and 0.92 × 10^−15^ m^3^/N.m for the micron at 300 °C. This was attributed to the increased oxidation of nano-sized particles resulting from their larger surface area.
- It was observed that abrasive wear accelerated by oxidative wear was the main wear mechanism for both sizes of WC at elevated temperatures and that the specific wear rates observed at a temperature of 600 °C were 75% lower for micron-sized particles (1.2 × 10^−15^ m^3^/N.m) than of nano-sized WC-9 wt.% Co (4.8 × 10^−15^ m^3^/N.m).
